# Annotating activation/inhibition relationships to protein-protein interactions using gene ontology relations

**DOI:** 10.1186/s12918-018-0535-4

**Published:** 2018-04-11

**Authors:** Soorin Yim, Hasun Yu, Dongjin Jang, Doheon Lee

**Affiliations:** 1 Department of Bio and Brain Engineering, KAIST, 291 Daehak-ro, Yuseong-gu, Daejeon, 34141 Republic of Korea; 2 Bio-Synergy Research Center, 291 Daehak-ro, Yuseong-gu, Daejeon, 34141 Republic of Korea

**Keywords:** Protein-protein interaction, Activation, Inhibition, Gene ontology

## Abstract

**Background:**

Signaling pathways can be reconstructed by identifying ‘effect types’ (i.e. activation/inhibition) of protein-protein interactions (PPIs). Effect types are composed of ‘directions’ (i.e. upstream/downstream) and ‘signs’ (i.e. positive/negative), thereby requiring directions as well as signs of PPIs to predict signaling events from PPI networks. Here, we propose a computational method for systemically annotating effect types to PPIs using relations between functional information of proteins.

**Results:**

We used *regulates*, *positively regulates*, and *negatively regulates* relations in Gene Ontology (GO) to predict directions and signs of PPIs. These relations indicate both directions and signs between GO terms so that we can project directions and signs between relevant GO terms to PPIs. Independent test results showed that our method is effective for predicting both directions and signs of PPIs. Moreover, our method outperformed a previous GO-based method that did not consider the relations between GO terms. We annotated effect types to human PPIs and validated several highly confident effect types against literature. The annotated human PPIs are available in Additional file 2 to aid signaling pathway reconstruction and network biology research.

**Conclusions:**

We annotated effect types to PPIs by using *regulates*, *positively regulates*, and *negatively regulates* relations in GO. We demonstrated that those relations are effective for predicting not only signs, but also directions of PPIs. The usefulness of those relations suggests their potential applications to other types of interactions such as protein-DNA interactions.

**Electronic supplementary material:**

The online version of this article (10.1186/s12918-018-0535-4) contains supplementary material, which is available to authorized users.

## Background

A cell reacts to stimuli through signaling pathways, in which proteins physically interact with each other to transmit signals. Those signals propagate inside a cell, causing various responses such as cell proliferation and differentiation [1–4]. Abnormal signal transduction triggers aberrant biological processes that might result in diseases such as cancer [2–5]. To understand how such signals flow, various high-throughput experiments have been developed to detect protein-protein interactions (PPIs) such as yeast two hybrid and affinity purification-mass spectroscopy [6]. Even though such high-throughput experiments can determine whether two proteins bind to each other or not, they are not sufficient for reconstructing signaling pathways.

To reconstruct signaling pathways from PPI networks, we need to know two aspects of PPIs: ‘directions’, and ‘signs’. Directions of PPIs represent upstream/downstream relationships, indicating the direction of signal flow. Signs of PPIs represent whether the interactions have positive effects or negative effects. By combining directions with signs, we can define activation/inhibition relationships of PPIs, which we call ‘effect types’.

Effect types are indispensable for not only reconstructing signaling pathways, but also other research areas such as network pharmacology [7, 8]. Without directions, we cannot know causality. This leads to many false positive results that arise from mistaking an effect as a cause [8]. Without signs, we cannot distinguish whether a result is desirable or harmful. For example, when signs are unavailable for drug-disease associations, we cannot differentiate whether a drug cures a disease, or causes a disease as a side effect [9].

Despite effect types are important, no experimental method is available that determines effect types of PPIs in a high-throughput way. Satisfying this need, several computational methods have been proposed to predict signs of PPIs systemically [10–12]. Based on data they used, previous works can be categorized into phenotype-based methods [10, 11] and a Gene Ontology (GO) [13]-based method [12]. Phenotype-based methods used RNA interference (RNAi) screening to identify phenotypes that were affected by a gene knockdown. Then, they predicted signs of PPIs based on the hypothesis that proteins resulting in similar phenotypes would interact positively [10, 11]. Even though they were effective, they have two limitations. Firstly, they ignored directions even though their aim was predicting effect types. Secondly, predicted signs cannot be generically applied to human PPIs. Because conducting RNAi screening for all proteins is experimentally expensive, they applied their method to smaller *Drosophila melanogaster* [10], or HeLa cells [11].

To overcome these limitations, a recent method utilized GO, which is more directly related to proteins [12]. Their hypothesis was that proteins with similar GO annotations would interact positively. They used GO terms as features for representing PPIs, and trained L2-regularized logistic regression model. Even though they improved the performance by using more direct data, they have mainly three limitations. Firstly, still they did not consider direction, leaving the causality between two proteins unknown. Secondly, similar GO annotations not necessarily means two proteins interact positively. In either positive PPIs or negative PPIs, two proteins interact with each other in any case. Therefore, negatively interacting proteins might also participate in the same biological process or have similar molecular function. In fact, the exactly same feature encoding was used for predicting whether two proteins interact or not, treating positive PPIs and negative PPIs equally [13]. Thirdly, they did not consider GO relations. However, GO has *positively regulates* and *negatively regulates* relations, which indicate signs between GO terms. These relations might help to differentiate negative PPIs in which one protein *negatively regulates* a biological process in which the other protein participates. Moreover, those relations indicate directions between GO terms, suggesting their potential use in predicting effect types of molecular interactions.

Here, we propose a method for annotating directions as well as signs to PPIs. We hypothesized that directions and signs between GO terms, represented by *regulates*, *positively regulates*, and *negatively regulates* relations, can be used for predicting directions and signs of PPIs. The rationale behind this hypothesis is as follows. Let us assume that protein p1 and p2 interact with each other, and there is a significant tendency in which GO terms involving p1 *positively regulates* GO terms involving p2. Since protein p1 and p2 interact with each other, the tendency of positive regulation might result from activation of p2 by p1. Based on this hypothesis, we predicted directions of undirected, unsigned PPIs first. Then, we predicted sign for each directed, unsigned PPI. PPIs were represented by features that were generated from *regulates*, *positively regulates*, and *negatively regulates* relations. Then, we trained logistic regression models for predicting directions and signs. Independent test results demonstrated that our method outperforms previous GO-based method, especially for negative PPIs. In addition, we annotated effect types to human PPIs and validated highly confident predictions against literature.

## Methods

### Method overview

**Fig. 1:**
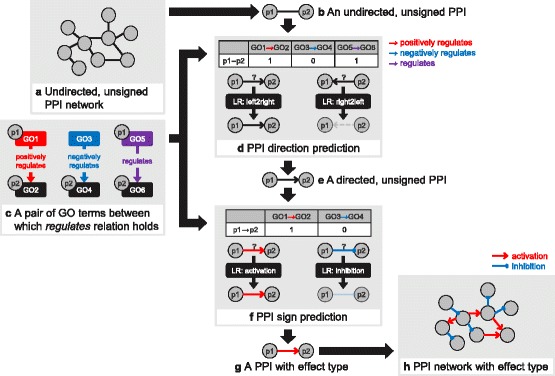
Method overview. **a** Input was an undirected, unsigned PPI network. **b**, **c**, **d** For each undirected, unsigned PPI, direction was predicted. We trained two logistic regression models that predicted whether the signal can flow in left-to-right direction and right-to-left direction, respectively. Feature vector was composed of pairs of GO terms between which *regulates*, *positively regulates*, or *negatively regulates* relation holds. **e** A directed, unsigned PPI was obtained as a result of direction prediction. **f** A sign of PPI was predicted for each directed PPI. We trained two logistic regression models that predicted whether a directed PPI can act as activation or inhibition, respectively. Feature vector was composed of a pair of GO terms between which *positively regulates* or *negatively regulates* relation holds. **g**, **h** As a result of direction prediction and sign prediction, we annotated effect types to PPI network. Abbreviations: GO, gene ontology; PPI, protein-protein interaction; LR, logistic regression

The overall method for annotating effect types to PPIs is illustrated in Fig. 1. The input was an undirected, unsigned PPI network. For each undirected, unsigned PPI, we predicted its direction first. We trained two logistic regression models that predicted whether a signal can flow in left-to-right direction and right-to-left direction, respectively. The two models shared the same feature vectors, which were composed of pairs of GO terms between which *regulates*, *positively regulates*, or *negatively regulates* relation hold. By combining outputs of these two models, we decided final direction of a PPI as one of ‘left-to-right’, ‘right-to-left’, and ‘bi-directional’. Then, we predicted sign for each directed, unsigned PPI. If the PPI is bi-directional, we predicted each sign for both directions. For predicting signs, we trained two logistic regression models that predicted whether a directed PPI can act as activation and inhibition, respectively. The two models shared an identical feature vector, which was composed of pairs of GO terms between which *positively regulates* or *negatively regulates* relation hold. By combining outputs of these two models, we decided final effect type as one of the followings: ‘activation’, ‘inhibition’, ‘activation&inhibition’, and ‘affect’. As a result, we obtained PPIs with effect type.

### Dataset

#### PPI dataset

We collected three PPI datasets: a training set, an independent test set, and a prediction set. To gather reliable datasets, we applied following policies to all datasets: (1) We collected human PPIs only. (2) We removed functional associations and self-interactions. (3) We collected PPIs only when at least one *regulates* relation holds between GO terms to which constituent proteins are annotated. (4) We mapped protein families to their members. (5) We integrated multiple instances of the same PPI to remove redundancy.

For the training set and the independent test set, we determined directions for each protein pair, and then determined sign for each directed PPI. For proteins protein 1 (p1) and protein 2 (p2), if a signal can flow in only one direction, for example from p1 to p2, the direction is ‘uni-directional’. On the other hand, if the signal can flow in both directions depending on a context, the direction is ‘bi-directional’. Then, we determined sign for each directed PPI. If a PPI is bi-directional, we determined sign for each direction independently. The same directed PPI can act as both activation and inhibition, depending on a context. For example, naked cuticle (NKD) binds to dishevelled segment polarity protein (DVL). This PPI acts as a switch from canonical Wnt signaling pathway to planar cell polarity (PCP) Wnt signaling pathway [14]. This means that NKD inhibits DVL in the aspect of canonical Wnt signaling pathway, whereas NKD activates DVL in the aspect of PCP Wnt signaling pathway. To deal with such context dependency, we categorized effect types of PPIs into four classes: ‘activation’, ‘inhibition’, both activation and inhibition are possible depending on a context (‘activation&inhibition’), and neither activation nor inhibition (‘affect’).

We collected PPIs with known effect types as a training set from Kyoto Encyclopedia of Genes and Genomes (KEGG) pathway [15]. KEGG is a manually curated database for pathways, and contains the largest number of PPIs whose effect types are known. Following the policies, we collected ‘PPrels’ whose subtypes were one of the followings: activation, inhibition, phosphorylation, dephosphorylation, glycosylation, and methylation.

We gathered another set of PPIs with known effect types as an independent test set from Search Tool for the Retrieval of Interacting Genes/Proteins (STRING) [16]. STRING is an integrated database for protein-protein associations, including functional and inferred associations. To secure reliable PPIs, we collected PPIs that were experimentally validated. Moreover, we used PPIs whose scores were higher than 800 out of 1000, which resulted in about 1.28% of the PPIs available in STRING. In addition, we removed PPIs that were in the training set.

We collected prediction set from Biological General Repository for Interaction Datasets (BioGRID), whose effect types were previously unknown and predicted by our method [17]. We collected multi-validated PPIs that were validated in at least two experimental systems or two publications. We removed PPIs that were in the training set or the independent test set. As a result, we obtained 20,192 PPIs as a training set, 3420 PPIs as an independent test set, and 28,742 PPIs as a prediction set as shown in Table 1.

**Table 1:** PPI dataset statistics

Direction					
	Uni-directional	Bi-directional		Total	
KEGG	20,078	114		20,192	
STRING	3110	310		3420	
BioGRID	–	–		28,742	
Effect type					
	Activation	Inhibition	Activation& inhibition	Affect	Total
KEGG	10,603	4548	207	447	15,805
STRING	2732	466	133	13	3344
BioGRID	–	–	–	–	–

#### GO dataset

We collected ontologies and GO annotations from GO [18]. We defined a concept of ‘regulators’ and ‘regulatees’ for GO terms. A regulator is a GO term that *regulates* another GO term. If it *positively regulates* another GO term, then it is a positive regulator, whereas it is a negative regulator if it *negatively regulates* one. Hereinafter we collectively refer to *regulates*, *positively regulates*, and *negatively regulates* as (*positively*/*negatively*) *regulates* when any of them are applicable. A regulatee is a GO term that is (positively/negatively) regulated by another GO term. For example, ‘chromatin silencing’ *negatively regulates* ‘transcription, DNA-templated’. Therefore, ‘chromatin silencing’ is a negative regulator whereas ‘transcription, DNA-templated’ is a regulatee.

To find all (positive/negative) regulators, we composed GO relations to form a composite relation, such that

1 $$ \mathrm{relation}\ 1\circ \mathrm{relation}\ 2\to \mathrm{composite}\ \mathrm{relation}.\kern6.75em $$

**Fig. 2:**
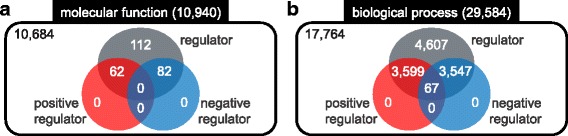
The statistics of GO terms. **a** Among 10,940 molecular function terms, 256 terms (2.3%) were regulators. **b** There were 11,820 (40.0%) regulators among 29,584 biological process terms, among which 67 terms were both positive and negative regulator

For instance, composing *is a* with *positively regulates* becomes *positively regulates*. Since ‘actin nucleation’ *is a* ‘positive regulation of actin filament polymerization’, which *positively regulates* ‘actin filament polymerization’, ‘actin nucleation’ becomes a positive regulator. This is called ‘relation reasoning’, and all possible composite relations are listed in Additional file 1: Table S1. We iteratively applied relation reasoning to find all (positive/negative) regulators, thereby increase coverage of our method. Hereinafter, we do not differentiate whether a regulator directly regulates regulatee, or indirectly regulates by a composite relation. The statistics of GO terms are shown in Fig. 2. Two hundred fifty six molecular function terms were regulators among 10,940 molecular function terms. For biological process terms, 11,820 terms were regulators out of 29,584 biological process terms.

### Feature generation for representing PPIs

**Fig. 3:**
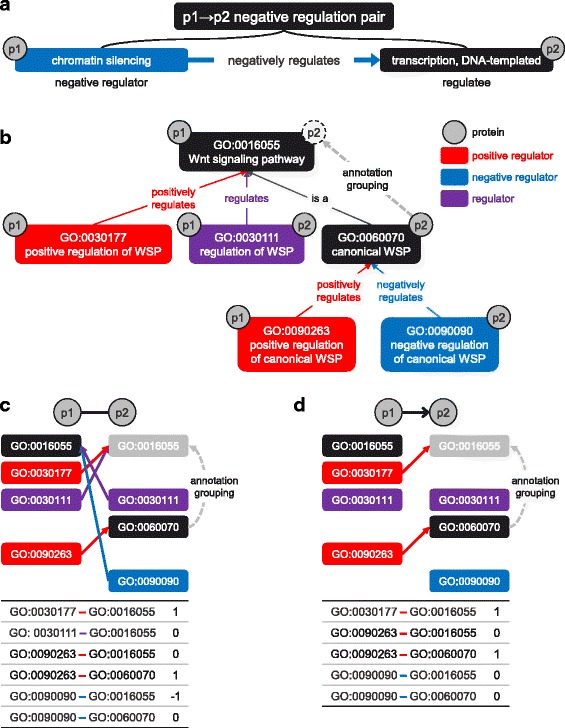
Feature generation for directions and signs. **a** ‘chromatin silencing’ *negatively regulates* ‘transcription, DNA-templated’. Thus, ‘chromatin silencing’ is negative regulator whereas ‘transcription, DNA-templated’ is a regulatee. If protein p1 and p2 is annotated to ‘chromatin silencing’ and ‘transcription, DNA-templated’ respectively, the two GO terms compose a p1 → p2 negative regulation pair, which represents direction and sign between two GO terms. **b** Feature generation procedures are explained with a toy example related to Wnt signaling pathway. There are four regulators (GO:0030177, GO:0030111, GO:0090263, GO:0090090), among which two are positive regulators (GO:0030177, GO:0090263) and one is a negative regulator (GO:0090090). Protein p1 is annotated to four GO terms (GO:0016055, GO:0030177, GO:0030111, GO:0090263), whereas protein p2 is annotated to three GO terms (GO:0030111, GO:0060070, GO:0090090). Protein p2 is not directly annotated to ‘Wnt signaling pathway’, but to ‘canonical Wnt signaling pathway’. Nonetheless, since ‘canonical Wnt signaling pathway’ *is a* ‘Wnt signaling pathway’, protein p2 is related to ‘Wnt signaling pathway’. **c** For GO1 and GO2 to which protein p1 and p2 are annotated respectively, we determined whether GO1 (*positively*/*negatively*) *regulates* GO2. If it did, GO1 and GO2 became a p1 → p2 (positive/negative) regulation pair. If it did not, we determined whether GO1 (*positively*/*negatively*) *regulates* any ancestors of GO2. Then, GO1 and the most specific ancestor of GO2 became a p1 → p2 (positive/negative) regulation pair. That way, we found (positive/negative) regulation pairs for p1 → p2, and p2 → p1 direction. To represent PPIs, we used regulation pairs as features. For directions, directions of regulation pairs were encoded as feature values. p1 → p2 (positive/negative) regulation pairs had the value of one, whereas p2 → p1 (positive/negative) regulation pairs had − 1. **d** For signs, signs of regulation pairs were encoded as feature values. p1 → p2 positive regulation pairs had the value of one, whereas p1 → p2 negative regulation pairs had − 1. Abbreviations: GO, gene ontology; WSP: Wnt signaling pathway

To encode the directions and signs between GO terms, we defined the concept of a p1 → p2 (positive/negative) regulation pair. A p1 → p2 (positive/negative) regulation pair is a pair of a (positive/negative) regulator and a corresponding regulatee, in which protein p1 is annotated to the (positive/negative) regulator and protein p2 is annotated to the regulatee. For example, if protein p1 is annotated to ‘chromatin silencing’ and protein p2 is annotated to ‘transcription, DNA-templated’, then ‘chromatin silencing’ and ‘transcription, DNA-templated’ constitute a p1 → p2 negative regulation pair as depicted in Fig. 3a.

A p1 → p2 (positive/negative) regulation pair indicates direction and sign between GO terms. We projected such directions and signs between GO terms to PPIs, by using each p1 → p2 (positive/negative) regulation pair as a feature for representing PPIs. We describe feature generation procedure with a toy example illustrated in Fig. 3b, which is a subset of GO terms related to Wnt signaling pathway.

Features were generated by following procedures. Firstly, we collected all GO terms to which proteins were annotated. In our toy example, protein p1 is annotated to four GO terms: ‘Wnt signaling pathway’, ‘positive regulation of Wnt signaling pathway’, ‘regulation of Wnt signaling pathway’, and ‘positive regulation of canonical Wnt signaling pathway’. On the other hand, protein p2 is annotated to three GO terms: ‘regulation of Wnt signaling pathway’, ‘canonical Wnt signaling pathway’, and ‘negative regulation of canonical Wnt signaling pathway’.

Secondly, for all possible pairs of GO1 and GO2 in which p1 is annotated to GO1 and p2 is annotated to GO2, we determined whether GO1 (*positively*/*negatively*) *regulates* GO2. If it did, then we regarded GO1 and GO2 as a p1 → p2 (positive/negative) regulation pair. However, in many cases, GO1 did not (*positively*/*negatively*) *regulate* GO2 itself. In such cases, we regarded that if p2 is annotated to GO2, then p2 is also annotated to ancestors of GO2 that have *is a* or *part of* relation with GO2. For example, despite p2 is not directly annotated to ‘Wnt signaling pathway’, we can say that p2 is related to ‘Wnt signaling pathway’ because p2 is annotated to ‘canonical Wnt signaling pathway’. This kind of extending GO annotations of a protein by using *is a* and *part of* relations in GO is called ‘annotation grouping’. To increase our coverage, if GO1 did not (*positively*/*negatively*) *regulates* GO2 itself, we applied annotating grouping and determined whether GO1 (*positively*/*negatively*) *regulates* any ancestors of GO2. If it did, then we found the most specific ancestor of GO2 with the highest information content that is regulated by GO1 [19]. Then, we regarded GO1 and the most specific ancestor of GO2 that is regulated by GO1 as a p1 → p2 (positive/negative) regulation pair. Since excessive annotation grouping might result in too high-dimensional feature vectors in which features are highly correlated, we did not applied annotation grouping when GO1 regulates GO2 itself. For the same reason, we used only the most specific ancestor of GO2, not all the ancestors.

For example, since p1 is annotated ‘positive regulation of canonical Wnt signaling pathway’ and p2 is annotated to ‘canonical Wnt signaling pathway’, the two GO terms form a p1 → p2 positive regulation pair. On the other hand, ‘positive regulation of Wnt signaling pathway’ does not regulate ‘canonical Wnt signaling pathway’. However, p2 is related to ‘Wnt signaling pathway’ when we apply annotation grouping. Thus, ‘positive regulation of Wnt signaling pathway’ and ‘Wnt signaling pathway’ constitute another p1 → p2 positive regulation pair.

We repeated the same procedure for determining whether GO2 (*positively*/*negatively*) *regulates* GO1 or its ancestors. As a result, we found six kinds of regulation pairs: p1 → p2 regulation pairs, p1 → p2 positive regulation pairs, p1 → p2 negative regulation pairs, p2 → p1 regulation pairs, p2 → p1 positive regulation pairs, and p2 → p1 negative regulation pairs. The six kinds of regulation pairs were used as features for predicting directions and signs of PPIs.

#### Feature generation for predicting directions of PPIs

For predicting directions of PPIs, we only considered the directions of (positive/negative) regulation pairs; whether it is from p1 to p2, or from p2 to p1. We did not differentiate regulation pairs, positive regulation pairs, and negative regulation pairs. The value of a (positive/negative) regulation pair GO1-GO2 is defined as:

2 $$ {\mathrm{f}}_{\mathrm{direction}}\left[\mathrm{GO}1-\mathrm{GO}2\right]=\left\{\begin{array}{c}1\kern1.25em \mathrm{if}\ \mathrm{GO}1-\mathrm{GO}2\ \mathrm{is}\ \mathrm{a}\ \mathrm{p}1\to \mathrm{p}2\ \left(\mathrm{positive}/\mathrm{negative}\right)\ \mathrm{regulation}\ \mathrm{p}\mathrm{a}\mathrm{ir},\mathrm{exclusively}\\ {}-1\ \mathrm{if}\ \mathrm{GO}1-\mathrm{GO}2\ \mathrm{is}\ \mathrm{a}\ \mathrm{p}2\to \mathrm{p}1\ \left(\mathrm{positive}/\mathrm{negative}\right)\ \mathrm{regulation}\ \mathrm{p}\mathrm{a}\mathrm{ir},\mathrm{exclusively}\\ {}0\kern31.75em \mathrm{otherwise}\end{array}\right. $$

In our toy example, since ‘positive regulation of canonical Wnt signaling pathway’ and ‘canonical Wnt signaling pathway’ constitute a p1 → p2 positive regulation pair, but not a p2 → p1 positive regulation pair, it has the value of one. On the other hand, ‘negative regulation of canonical Wnt signaling pathway’ and ‘Wnt signaling pathway’ form a p2 → p1 negative regulation pair, exclusively. Thus, it has the value of − 1. If the direction of a (positive/negative) regulation pair is both from p1 to p2 and from p2 to p1, then the feature value is zero. In the toy example, since p1 → p2 (positive/negative) regulation pairs outnumber p2 → p1 (positive/negative) regulation pairs, the direction of PPI is more likely to be from p1 to p2. We removed (positive/negative) regulation pairs that were not used in the training set and the number of features for direction was 37,617.

#### Feature generation for predicting signs of PPIs

Feature generation for predicting signs of PPIs are similar to that for directions; We used each regulation pair as a feature. However, there are also some differences: (1) Since we predicted sign for a directed PPI, we used regulation pairs whose directions were consistent with the direction of the PPI. (2) Since simple regulation pairs are uninformative for predicting signs, we used positive regulation pairs and negative regulation pairs only. (3) We removed regulation pairs that is both positive and negative. For example, ‘cell cycle switching, mitotic to meiotic cell cycle’ *positively regulates* ‘meiotic cell cycle’ and *negatively regulates* ‘mitotic cell cycle’. Thus, ‘cell cycle switching, mitotic to meiotic cell cycle’ both *positively* and *negatively regulates* ‘cell cycle’. We removed those regulation pairs since they are meaningless. (4) Compared to a feature vector for direction, which signifies the direction between GO terms, a feature vector for sign indicates signs. For predicting sign of a directed PPI p1 → p2, the value of a positive/negative regulation pair GO1-GO2 is defined as:

3 $$ {\mathrm{f}}_{\mathrm{sign}}\left[\mathrm{GO}1-\mathrm{GO}2\right]=\left\{\begin{array}{c}1\kern1.75em \mathrm{if}\ \mathrm{GO}1-\mathrm{GO}2\ \mathrm{is}\ \mathrm{a}\ \mathrm{p}1\to \mathrm{p}2\ \mathrm{p}\mathrm{ositive}\ \mathrm{regulation}\ \mathrm{p}\mathrm{a}\mathrm{ir},\mathrm{exclusively}\\ {}-1\kern0.75em \mathrm{if}\ \mathrm{GO}1-\mathrm{GO}2\ \mathrm{is}\ \mathrm{a}\ \mathrm{p}1\to \mathrm{p}2\ \mathrm{negative}\ \mathrm{regulation}\ \mathrm{p}\mathrm{a}\mathrm{ir},\mathrm{exclusively}\\ {}0\kern27em \mathrm{otherwise}\end{array}\right. $$

In our toy example, since ‘positive regulation of canonical Wnt signaling pathway’ and ‘canonical Wnt signaling pathway’ is a p1 → p2 positive regulation pair, it has the value of one. On the other hand, since ‘negative regulation of canonical Wnt signaling pathway’ and ‘Wnt signaling pathway’ is a p2 → p1 negative regulation pair, not p1 → p2, it has the value of zero. In the toy example, since p1 → p2 positive regulation pairs outnumber p1 → p2 negative regulation pairs, p1 more likely activates p2 rather than inhibits p2. We removed regulation pairs that were not used in the training set. The number of features for sign was 20,077.

### Model generation for performance evaluation

We used L2-regularized logistic regression for predicting directions and signs of PPIs. We used logistic regression because it is interpretable [20]. Moreover, L2-regularization reduces overfitting that might be caused by high-dimensionality of feature vectors. As shown in Table 1, we had much more activating PPIs than inhibiting ones. To overcome this imbalance, we adopted cost-sensitive learning in which class-weight was inversely proportional to the class frequency [21].

#### Model generation for predicting directions of PPIs

We trained two L2-regularized logistic regression models that shared the same feature vectors for predicting directions of undirected, unsigned PPIs; they predicted whether a signal could flow in left-to-right direction and right-to-left direction, respectively. By combining outputs from the two models, we determined final directions as one of the followings: ‘left-to-right’, ‘right-to-left’, or ‘bi-directional’. For example, if a signal is predicted to be able to flow in ‘left-to-right’ direction, but not in ‘right-to-left’ direction, then the final direction of PPI is ‘left-to-right’. If a signal can flow in both direction, then the final direction of PPI is ‘bi-directional’. Instead of training one classifier that predicts three outcomes, we trained two classifiers separately because uni-directional PPIs highly outnumbered bi-directional ones as shown in Table 1. During the training and test phase, we randomly divided uni-directional PPIs into two equal-sized sets: left-to-right PPIs, and right-to-left PPIs.

#### Model generation for predicting signs of PPIs

For each directed, unsigned PPI, we predicted its effect type as one of ‘activation’, ‘inhibition’, ‘activation&inhibition’, and ‘affect’. Similar to directions, signs of PPIs were highly imbalanced; the number of ‘activation&inhibition’ and ‘affect’ class were very low. Thus, we trained two classifiers, rather than a single classifier that predicts four possible outcomes. The two classifiers shared the same feature vectors and predicted whether a directed PPI can act as activation and inhibition, respectively. Then, final effect types were determined by combining outputs of two classifiers. If a directed PPI can act as ‘activation’, but not as ‘inhibition’, then its effect type were determined as ‘activation’, and vice versa.

## Results and discussions

### Cross-validation performances

We applied our method to KEGG dataset, and conducted 10-fold cross-validation. In 10-fold cross validation, KEGG dataset is split into ten disjoint subsets. Then, we trained logistic regression models by using nine subsets, and tested the models on the remaining one subset. This procedure was repeated such that the models can be evaluated for each subset. The performance was obtained for each subset, and the mean performance is listed in Table 2. For directions, the performance of left-to-right and right-to-left classifiers were almost identical. F1-score and accuracy were as high as 0.89 for both left-to-right and right-to-left classifiers. Area under receiver operating characteristics (AUROC) and area under precision-recall curve (AUPRC) was 0.95 and 0.94 for both classifiers, respectively [see Additional file 1: Figure S1].

**Table 2:** Performance of classifiers for 10-fold cross validation

Classifier	Precision	Recall	F1-score	Accuracy
Direction				
Left2right	0.894 ± 0.025	0.890 ± 0.029	0.892 ± 0.027	0.892 ± 0.027
Right2left	0.893 ± 0.029	0.892 ± 0.023	0.892 ± 0.025	0.892 ± 0.026
Effect type				
Activation	0.923 ± 0.031	0.902 ± 0.059	0.912 ± 0.040	0.881 ± 0.050
Inhibition	0.791 ± 0.103	0.819 ± 0.088	0.800 ± 0.077	0.876 ± 0.052

The performance is shown as mean ± standard deviation

For signs, f1-score of activation and inhibition classifier were 0.91 and 0.80, respectively. F1-score of activation classifier was higher because activating PPIs outnumbered inhibiting ones. Accuracy of two classifiers were identical as 0.88. AUROC were 0.94 and 0.93 for activation and inhibition classifiers, respectively. Also, AUPRC were 0.96 and 0.89 for activation and inhibition classifiers, respectively [see Additional file 1: Figure S1].

### Independent test performances

To see how well our model generalizes to datasets from different sources, we conducted an independent test. In an independent test, we trained logistic regression models with KEGG dataset, and tested with STRING dataset. The performance for predicting directions and signs are listed in Table 3. The performance of left-to-right classifier and right-to-left classifier were similar. Accuracy of left-to-right classifier and right-to-left classifier were around 0.6 and 0.59, respectively. The AUROC of left-to-right classifier and right-to-left classifier were 0.64 and 0.63, respectively [see Additional file 1: Figure S2].

**Table 3:** Performance of classifiers for independent test

Classifier	Precision	Recall	F1-score	Accuracy
Direction				
Left2right	0.64	0.60	0.62	0.60
Right2left	0.64	0.58	0.61	0.59
Effect type				
Activation	0.91	0.71	0.79	0.69
Inhibition	0.29	0.49	0.37	0.69

The performance for predicting sign was higher than predicting directions. The accuracy of activation classifier and inhibition classifier were both 0.69. However, because the dataset was imbalanced, f1-score was much higher in activation classifier than inhibition classifier. AUROC of activation classifier and inhibition classifier were 0.67 and 0.63, respectively [see Additional file 1: Figure S2].

### Comparison with the previous GO-based method

To demonstrate the effectiveness of GO relations, we compared the performance of our method to the performance of the previous GO-based method that did not use GO relations. Since the previous work did not consider directions of PPIs, we slightly modified our PPI dataset and feature values for the comparison. For PPI datasets, we originally defined effect types for directed PPIs. Therefore, bi-directional PPIs have two effect types, one effect type for each direction. To compare performance with the previous work, we mapped effect types of directed PPIs to undirected PPIs by using ‘OR’ operation so that bi-directional PPIs have only one effect type. For an undirected PPI p1-p2, if either directed PPIs p1 → p2 or p2 → p1 can act as activation, then p1-p2 has ‘activation’ as its effect type. Likewise, if either p1 → p2 or p2 → p1 can act as ‘inhibition’, then p1-p2 has ‘inhibition’ as its effect type. The statistics of PPI dataset that were used for the performance comparison is listed in Table 4.

**Table 4:** Statistics of PPI datasets that were used for comparison with previous GO-based method

		Activation	Inhibition	Activation&inhibition	Affect	Total
KEGG	Previous work	14,054	5478	382	552	20,466
	Our work	12,309	4787	369	517	17,982
STRING	Previous work	2849	465	152	15	3481
	Our work	2729	455	152	15	3351

The previous GO-based method covers all PPIs whose constituent proteins are annotated to at least one GO term. However, our method requires at least one positive/negative regulation pair to predict effect type, thereby decreasing our coverage

For feature generation, when we predict effect type of a directed PPI p1 → p2, originally we considered p1 → p2 positive/negative regulation pairs only. p2 → p1 positive/negative regulation pairs were omitted because the direction between GO terms were inconsistent with the direction of the PPI. However, since the directions were not considered in the performance comparison, we used both p1 → p2 positive/negative regulation pairs and p2 → p1 positive/negative regulation pairs.

**Fig. 4:**
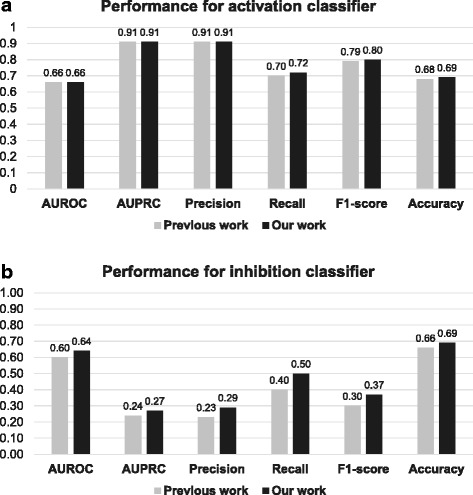
Performance comparison with the previous GO-based method. We conducted independent test to compare performance with the previous GO-based method that did not consider GO relations. We compared the performance of activation and inhibition classifiers in terms of AUROC, AUPRC, precision, recall, f1-score, and accuracy. Since the previous work did not consider directions, performance for predicting signs was compared. **a** For predicting activation, our work performed equally, or slightly better than the previous work. **b** For predicting inhibition, our work outperformed the previous work by all metrics. These results show that overlap or difference between GO annotations of two proteins are not sufficient discriminating factor for signs of PPIs, given that inhibiting PPIs also participate in the same biological process. Moreover, *positively regulates* and *negatively regulates* relations in GO can be used for enhancing performance for predicting signs of PPIs. Abbreviations: AUROC, area under receiver operating characteristics; AUPRC, area under precision-recall curve

We conducted independent test for the previous work and our work. In the independent test, logistic regression models were trained with KEGG dataset, and tested with STRING dataset. The independent test results are shown in Fig. 4. For activation classifier, the performance was identical or slightly better in our method. On the other hand, in inhibition classifier, our method outperformed the previous work, especially in terms of recall. These results show that our method solved the second and third limitations of the previous work that we mentioned. The second limitation was that even if one protein inhibits another protein, the two proteins might share the same GO terms because they interact with each other. This may result in similar feature vector between activating PPIs and inhibiting PPIs in the previous work, suggesting that simply considering whether two proteins share the same GO terms are not sufficient for predicting signs of PPIs. We solved this problem by using *positively regulates* and *negatively regulates* relations in GO, not using which was the third limitation. Enhanced performance demonstrates that those relations help to predict signs of PPIs, especially for inhibiting ones.

Even though our method outperformed the previous GO-based method, our method has one drawback; slightly lower coverage. Since we need at least one positive/negative regulation pair for predicting effect types, we covered 89% of PPIs that were covered by the previous method, as shown in Table 4.

### Annotation of effect types to human PPIs

We applied our method to the prediction set. We validated top five most confident predictions against literature, each for activation and inhibition. We were able to find five publications supporting our prediction, as shown in Table 5.

**Table 5:** Literature validation of top five activating and inhibiting PPIs from effect type-annotated human PPIs

Protein1	Predicted effect type	Protein2	Supporting evidence
KIT	Activation	STAT1	[24]
NRG1	Activation	LIMK1	[25]
ADAM17	Activation	MAPK1	
ABL1	Activation	BRCA1	[26]
FGF2	Activation	RPS19	
AXIN1	Inhibition	GSK3A	
GRB10	Inhibition	IRS1	[27]
MAD7	Inhibition	SMAD4	
GRB10	Inhibition	AKT1	[28]
TLE1	Inhibition	LEF1	

### Case study

**Fig. 5:**
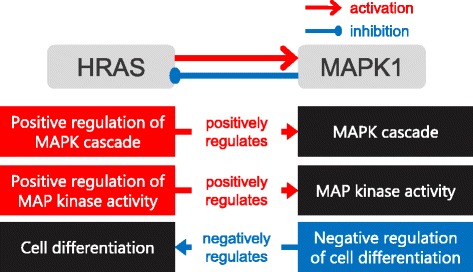
A case study for PPI where one direction is activation and the other is inhibition. HRAS activates MAPK1 in axon guidance, whereas MAPK1 inhibits HRAS in neutrophin signaling pathway. Both effect types were correctly predicted in 10-fold cross validation, showing that our method can predict sign for each direction independently. Positive/negative regulation pairs that contributed to the sign prediction are shown in the figure. Interestingly, ‘negative regulation of cell differentiation’ and ‘cell differentiation’ were used to predict the inhibition, which is related to the function of neutrophin signaling pathway. Abbreviations: HRAS, hras proto-oncogene, GTPase; MAPK1, mitogen-activated protein kinase 1

To demonstrate that our method can predict effect types of PPIs even when one direction is activation and the other direction is inhibition, we conducted a case study. Hras proto-oncogene, GTPase (HRAS) activates mitogen-activated protein kinase 1 (MAPK1) in axon guidance, a process in which axon growth cone migrates in specific direction. On the other hand, MAPK1 inhibits HRAS in neutrophin signaling pathway. In 10-fold cross-validation, our method correctly predicted effect types of two directions, even when one direction is activation and the other is inhibition. This means that since we predict signs for each directed PPI, and use positive/negative regulation pairs only when their directions are consistent with the directions of PPIs, our method is able to predict effect types of PPIs independently for both directions. Regulation pairs used for the prediction is illustrated in Fig. 5. For predicting that HRAS activates MAPK1, ‘positive regulation of MAPK cascade’ and ‘positive regulation of MAP kinase activity’ were used. On the other hand, ‘negative regulation of cell differentiation’ was used for predicting that MAPK1 inhibits HRAS, which is related to the function of neutrophin signaling pathway. This means that some regulation pairs reflect the context in which the PPI occurs.

## Conclusions

In this work, we predicted effect types of PPIs by using *regulates*, *positively regulates*, and *negatively regulates* relations in GO. We hypothesized that directions and signs between GO terms can be used to predict directions and signs of PPIs. For an undirected, unsigned PPI, we predicted its direction first. We trained two logistic regression models to predict whether a signal can flow in left-to-right direction and right-to-left direction, respectively. The directions of (*positively*/*negatively*) *regulates* relations were encoded as features for representing direction of PPI. Then, we predicted sign for each directed PPI, thereby predicting effect type of PPI. We also trained two logistic regression models for predicting whether a directed PPI can act as activation and inhibition, respectively. We represented a directed PPI with features whose values were signs of *positively*/*negatively regulates* relations. As a result, we annotated effect types to PPIs, thereby turning PPI network into a directed, signed graph.

Our contribution is two-fold. Firstly, we proposed a concept of p1 → p2 (positive/negative) regulation pair, which is effective for predicting directions, as well as signs of PPIs. This solves the limitation of previous works that were not able to predict directions of PPIs. Secondly, we demonstrated usefulness of (*positively*/*negatively*) *regulates* relations in GO. Up to date, most of GO-related works have used only *is a* and *part of* relations. In this work, we showed that (*positively*/*negatively*) *regulates* relations are effective for predicting directions and signs of PPIs, suggesting their extension to other types of interactions. For example, those relations might be used for predicting signs of protein-DNA interactions; whether a transcription factor activates or represses expression of a target gene.

Even though our work improved the performance for predicting signs of PPIs, we have some drawbacks. Since we need at least one positive/negative regulation pairs for predicting signs, our method has lower coverage than the previous GO-based method. We applied relation reasoning and annotation grouping to compensate low coverage of (*positively*/*negatively*) *regulates* relations, nevertheless, some PPIs were not covered. At this moment, our method did not consider specificity of GO terms. However, more specific GO terms have clearer meaning. This suggests that reflecting specificity of GO terms might improve our method. In addition, the performance of inhibition classifier was much lower than activation classifier since there were much more activating PPIs than inhibiting ones. This imbalance was so significant that cannot be perfectly corrected by cost-sensitive learning. We expect that accumulation of more inhibiting PPIs will enhance the performance in future.

To facilitate signaling pathway reconstruction and network biology research, we provided effect type-annotated human PPIs in Additional file 2. The annotated effect types turned PPI network into a directed signed graph, opening up opportunities for discovering new characteristics of PPI network or signaling pathways. For example, signs of PPIs can be used for measuring stability of PPI network [10, 22]. In addition, effect types of PPIs can be used for discovering novel regulators of signaling pathways [10, 23], and improving performance for predicting efficacies of drugs [8].

## Additional files

Additional file 1: Table S1. Lists all possible combinations of GO relations where relation reasoning can be applied. **Figure S1-S2.** shows ROC and PRC along with their area under the curves obtained from cross-validation, and independent test results. (PDF 799 kb)

Additional file 2: Effect type-annotated human PPIs. This file contains effect type-annotated human PPIs in .xls format. Each row is a triplet of (protein 1, effect type, protein 2), in which protein 1 is a upstream protein whereas protein 2 is a downstream protein. (XLS 1547 kb)

## Abbreviations

AUPRC: Area under precision-recall curve
AUROC: Area under receiver operating characteristics
DVL: Dishevelled segment polarity protein
GO: Gene ontology
HRAS: Hras proto-oncogene, GTPase
MAPK1: Mitogen-activated protein kinase 1
NKD: Naked cuticle
PCP: Planar cell polarity
PPI: Protein-protein interaction
RNAi: RNA interference
