# Supplementary material for: Annotating activation/inhibition relationships to protein-protein interactions using gene ontology relations
Source: BMC Syst Biol. 2018 Apr 11;12(Suppl 1):9. doi: 10.1186/s12918-018-0535-4 (PMC5907154; doi:10.1186/s12918-018-0535-4)
Supplement: Supplementary file 1 — Lists all possible combinations of GO relations where relation reasoning can be applied. Figure S1-S2. shows ROC and PRC along with their area under the curves obtained from cross-validation, and independent test results. (PDF 799 kb) [file 12918_2018_535_MOESM1_ESM.pdf]

Table S1. GO relation reasoning. This table lists all possible relation reasoning in GO such that $\text{relation 1} \circ \text{relation 2} \rightarrow \text{composite relation}$

**Relation 1**	**Relation 2**	**Composite relation**
Is a	Is a	Is a
Is a	Part of	Part of
Is a	Regulates	Regulates
Is a	Positively regulates	Positively regulates
Is a	Negatively regulates	Negatively regulates
Part of	Is a	Part of
Part of	Part of	Part of
Regulates	Is a	Regulates
Regulates	Part of	Regulates
Positively regulates	Is a	Positively regulates
Positively regulates	Part of	Regulates
Negatively regulates	Is a	Negatively regulates
Negatively regulates	Part of	Regulates

Figure S1. ROC and PRC for cross-validation results

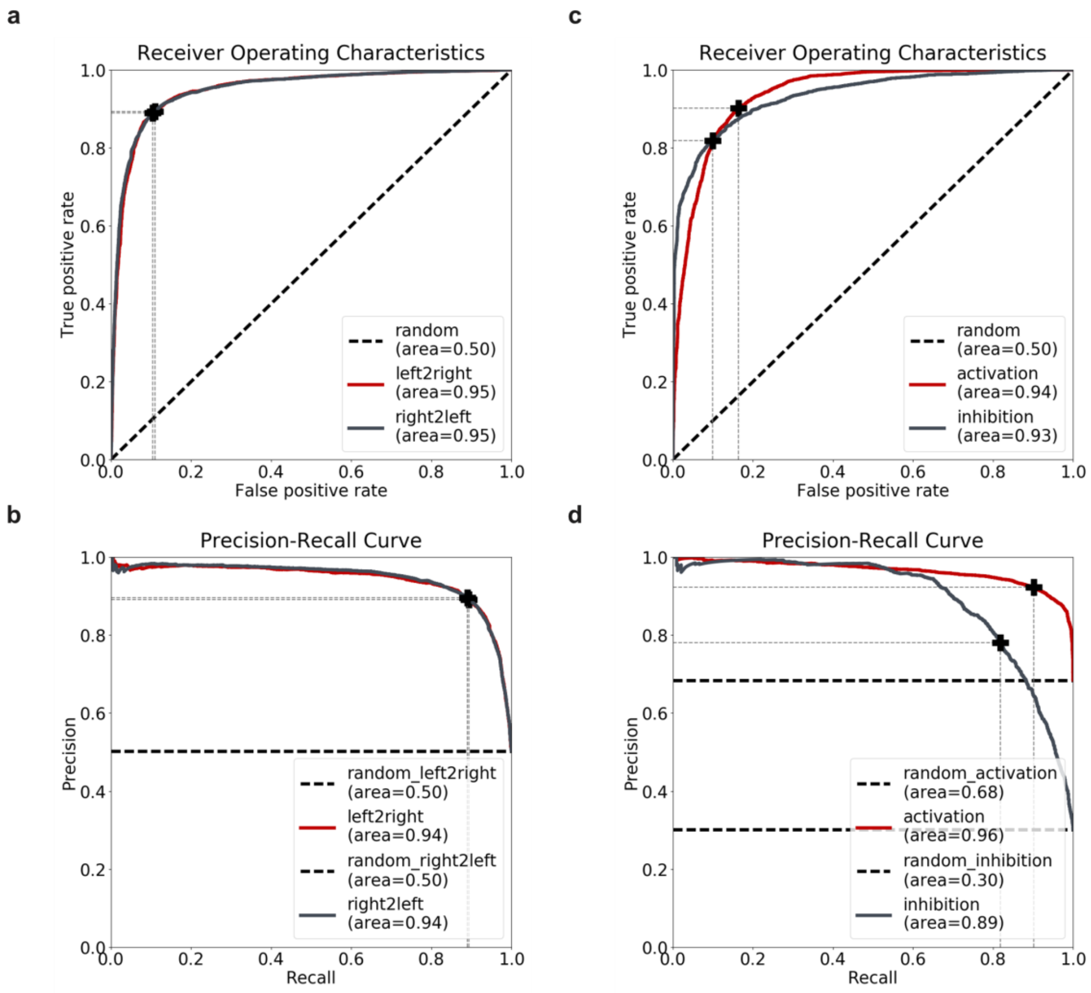

(a) ROC for direction classifiers. Left-to-right and right-to-left classifiers performed similarly with the equal AUROC of 0.95. (b) PRC for direction classifiers. Left-to-right and right-to-left classifiers performed similarly with the equal AUPRC of 0.94. (c) ROC for sign classifiers. Activation classifier was slightly better than inhibition classifier, with AUROC of 0.94 and 0.93, respectively. (d) PRC for sign classifiers. AUPRC of activation and inhibition classifiers were 0.96 and 0.89, respectively. A plus mark shows the performance of the threshold that was applied for the classification.

Figure S2. ROC and PRC for independent test results

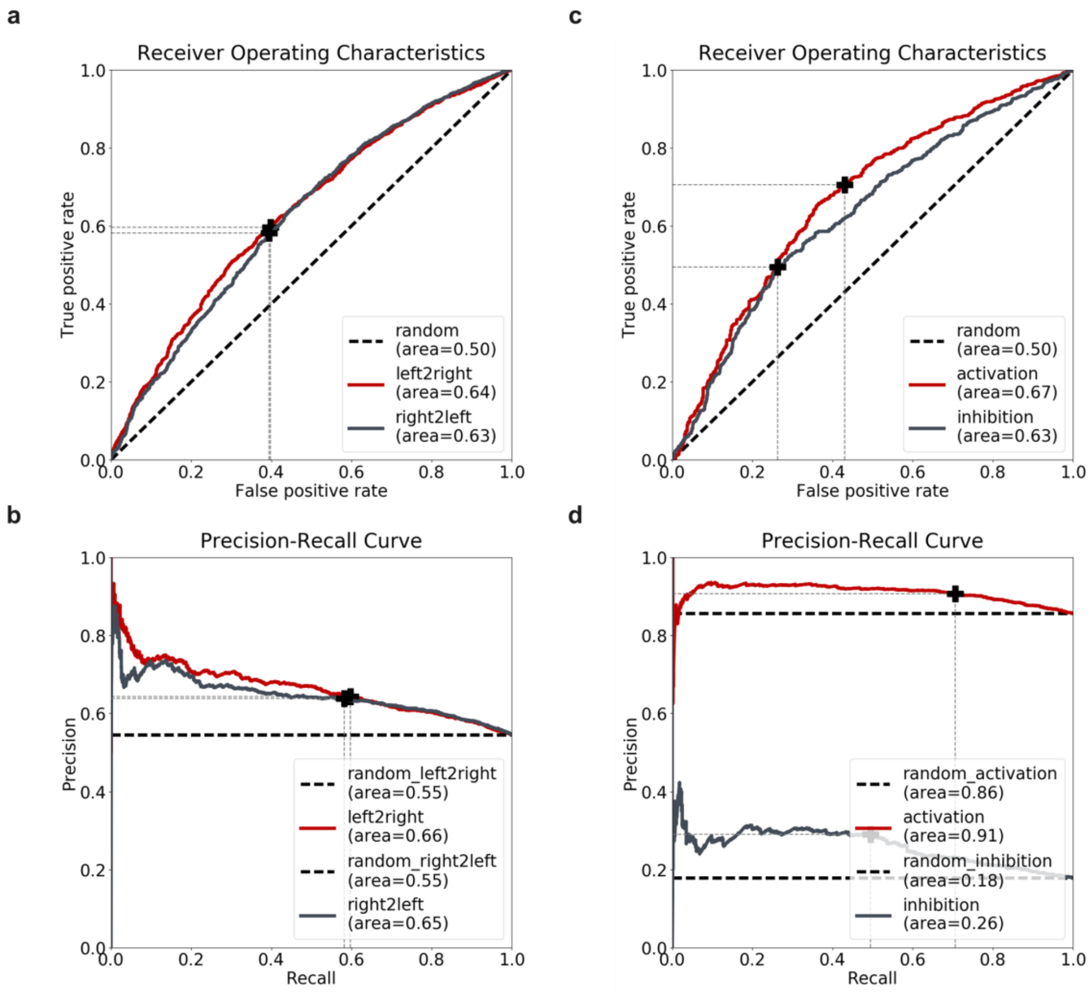

(a) ROC for direction classifiers. Left-to-right and right-to-left classifiers performed similarly with AUROC of 0.64 and 0.63, respectively. (b) PRC for direction classifiers. Left-to-right and right-to-left classifiers performed similarly with AUPRC of 0.66 and 0.65, respectively. (c) ROC for sign classifiers. Activation classifier performed better than inhibition classifier, with AUROC of 0.67 and 0.63, respectively. (d) PRC for sign classifiers. AUPRC of activation and inhibition classifiers were 0.91 and 0.26, respectively. A plus mark shows the performance of the threshold that was applied for the classification.
